# Beyond barriers, towards diversity: how hybrid student conferences can drive accessibility

**DOI:** 10.1242/bio.060290

**Published:** 2024-01-30

**Authors:** Janire Castellano Bueno, Alexandros Vezyrakis, Peter Xu, Christopher W. Miller

**Affiliations:** ^1^ Wild Animal Initiative, UK; ^2^ Newcastle University, UK; ^3^ Max Planck Institute for Evolutionary Biology, Plön, Germany; ^4^ University of Potsdam, Germany; ^5^ University of York, UK

**Keywords:** Accessibility, Animal behaviour, Early career researchers, Hybrid conferencing, Inclusive Conferencing, Student conferences

## Abstract

The third International Student Symposium on Animal Behaviour and Cognition (ISSABC) aimed to address barriers for early career researchers, hosting a conference both in-person and online at the Universidad Nacional Autónoma de México (UNAM). The conference, attended by 101 in-person and 79 virtual participants from 24 countries, featured 81 presentations, 29 posters, five plenary talks, three workshops, and a career development round-table discussion. A user-friendly website and digital platforms facilitated communication and real-time discussions between in-person and online participants. Transparent fund management, support from sponsors and societies, and sustainable practices ensured financial accountability and minimised environmental impact. The conference emphasised sustainability measures, including eco-friendly catering and local reusable mugs. Future organisers of similar events are encouraged to prioritise local representation, plan contingencies, select plenary speakers rigorously, and employ effective marketing. With this meeting review, we highlight how hybrid conferences like the third ISSABC, through innovative approaches and sustainable practices, enhance accessibility, inclusivity, and empower the next generation of scientists.

## Teaser

**Summary:** This Meeting Review presents conclusions from the third International Student Symposium on Animal Behaviour and Cognition (ISSABC), an international hybrid conference organised by ECRs that aimed to provide a larger, international stage for ECRs to present their research and network.

## Introduction

### Field

**Fig. 1.**
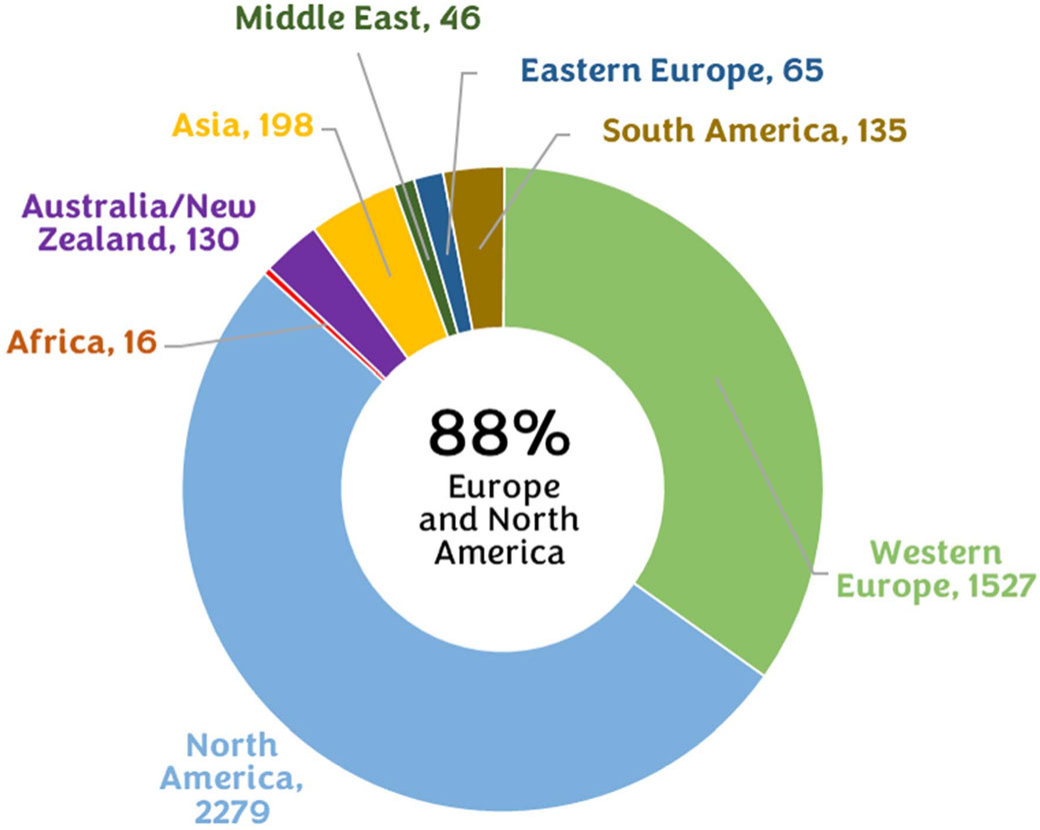
**Publication trends between 2001–2002 across the world for animal behaviour journals.** The proportion of total manuscripts by region, with values in parentheses representing absolute numbers of manuscripts. Adapted from Ord et al. (2005), which reflects where most of the institutions that fund this research are based.

Numerous large international conferences, smaller local meetings and specialised workshops have been offering opportunities for animal behaviour researchers to share their findings, network with peers and learn. Early career researchers (ECRs), in particular, have benefited from several newly established, mostly local, meetings that have created unique opportunities to present their research to an audience beyond their immediate research group. However, these meetings are still mostly restricted largely to the Global North [reflecting where most of the institutions that fund animal behaviour research are based (Ord et al., 2005), see Fig. 1], and present a substantial socioeconomic barrier for international audiences to attend. Here, we present conclusions from an international hybrid conference organised by ECRs that aimed to provide a larger, international stage for ECRs to present their research and network while removing the usual barriers that have existed historically in science.

### Barriers to traditional conferences

In-person conferences provide a platform for sharing latest research findings and methodologies, and receive immediate feedback (Raby and Madden, 2021). Additionally, in-person conferences foster networking opportunities, enabling participants to connect with peers, mentors, and potential collaborators in a more personal way that would be impossible through virtual means. Thus, in-person conferences contribute to a sense of community, knowledge dissemination, and collective advancement within the field.

In-person events are often hosted in the just a few geographical areas, introducing several challenges for travelling attendees. As these events are usually hosted in the Global North, attendees from the Global South (and even some areas of the Global North, e.g. Eastern Europe) are deterred from participating due to the requirement of travelling long distances, and the associated financial cost of doing so. This results in a lack of diversity in perspectives, as attendees often come from similar academic and cultural backgrounds, limiting the cross-cultural exchange of ideas and comprehensive understanding of animal behaviour across various ecosystems.

Students from low-income countries are amongst those worst affected by these issues. Financial constraints are a significant hurdle, as these events involve travel, accommodation, and conference registration costs. For students from the Global South, these costs can be particularly prohibitive due to salary and funding differences between Global North and Global South countries. The lack of institutional support from these regions further exacerbates these challenges. Without proper institutional backing in the form of financial assistance or experiences in networking, not only is students’ career development stunted, but their absence also contributes to the underrepresentation of diverse voices in the field.

Furthermore, attendees from outside the EU and the US often face obstacles when applying for visas to attend educational events. Challenges include strict requirements, uncertain processing times, geopolitical tensions, limited consular services, inflexible policies, and potential bias and discrimination, adding extra barriers to their participation in in-person conferences. Again, these obstacles are especially relevant for ECRs, who often have access to fewer additional funding opportunities and more legal obstacles to travel outside their home countries. As a result, ECRs from low-income countries often find it challenging to establish varied connections due to their limited presence at international gatherings.

Finally, language barriers pose additional challenges. With most conferences conducted in English, non-native speakers struggle to fully engage, understand complex concepts, and participate in discussions. Lack of confidence in their language skills further reduces their chances of even applying as they believe that, simply because of this language barrier, their chances of succeeding are low. This can hinder learning, networking, and collaboration opportunities, underscoring the need for multilingual support and inclusive communication strategies.

Recognising the above issues and working to establish partnerships, scholarships, or grants aimed specifically at supporting attendees from underrepresented regions can help bridge the gap and contribute to a more inclusive and globally diverse animal behaviour community.

### Objectives

Our objectives when organising the third ISSABC were to (1) create a conference made by students for students, (2) promote the study of animal behaviour, (3) promote justice, equality, diversity and inclusion and (4) promote sustainable conferencing practices. The conference was held on the 21st-23rd June, 2023 at UNAM. Using both in-person and online participation, the conference aimed to bring together animal behaviour students worldwide.

## Conference overview

The conference was organised by student volunteers with no institutional funding. A hybrid format was chosen to boost accessibility to students irrespective of location and financial means (Chou and Camerlink, 2021). The online platform (Zoom Video Communications, Inc., 2022) also served as a contingency plan in case unforeseen circumstances would make the in-person event impossible. Participants could present their work in-person or virtually, as Standard Talks (10 min), Flash Talks (3 min) or Posters. Online participants could pre-record their talk to mitigate issues with poor connectivity. These talks, as well as the plenary talks, were streamed on Zoom, and recordings were made available for registered participants to watch on the conference website. Posters were shown both at the conference and on the conference website. The software Discord (Discord, Inc., 2022) allowed the creation of individual online discussion fora for each talk and poster. Sustainable conferencing practices were applied where possible, including vegetarian and vegan lunches, reusable, locally produced mugs and no single-use plastic.

## Preparing the conference

### Team assembly

Initially, student volunteers formed a team for the third ISSABC. With past organisers advertising the call both in-person and on Twitter, a meeting engaged interested students, offering insights into roles. After the handover, the new committee promoted the opportunity at animal behaviour conferences and on social media.

Challenges in recruiting organisers included clarifying responsibilities, time commitment, and the non-material rewards that this experience can provide. The committee's connections facilitated collaboration with UNAM, providing event space, outreach, an official email, and designing the conference logo (Fig. 1; Supplementary Materials).

### Advertising

We used various advertising channels both for the promotion of the event for attendees as well as for sponsors and potential students interested in helping in the organisation. One encountered challenge was the difference in the use of social media depending on the country of origin and residence. As an international event with not enough representatives of the target countries in the organising committee, this was a challenge that had a significant impact on the final number of attendees. As an example, Facebook is routinely used in Mexico for disseminating information about academic events, while in the USA and UK Twitter is currently the main social media used by academics, and Facebook is never used for that audience.

In addition to social media, we collaborated with existing animal behaviour societies to share the information and invited all postgraduate students from UNAM individually by email using the tools on the faculty's website. To reach students from institutions across the world, we contacted relevant departments in various institutions directly through email and social media contacts, as well as in-person by the committee members at various international conferences the year before the third ISSABC.

### Sponsorship

As a conference held with no institutional support, we had to conduct an extensive sponsorship campaign to secure sufficient funds to run the conference and achieve our objectives of removing the financial barriers from those attending. This included (1) finding a venue/hosting institution, (2) reaching out to private sponsors, (3) reaching out to scientific societies and (4) applying for grants. These were accomplished using various strategies:

(1) **Venue:** Our primary aim for the third ISSABC was hosting a meeting beyond traditional Global North settings. UNAM kindly agreed to act as a host institution, by providing the venue (amphitheatre, two rooms, and a large break/poster area in the Amoxcalli building at the UNAM campus) and designing the conference logo on a non-profit basis.
(2) **Private Sponsors:** Contacted more than 50 potential sponsors, especially those relevant to animal behaviour, yielded collaboration with seven sponsors. Four offered financial support, while three provided prizes. Sponsorship tiers offered varying exposure, from abstract book mentions to conference booths.
(3) **Societies:** Contacted global scientific societies for both monetary support and advertising to their members. Multiple societies shared conference news on social media and newsletters, with two offering monetary support for student grants and plenary speakers.
(4) **Grants:** Applied for five grants, secured one for the conference, one for a plenary speaker's travel, and one for a committee member's partial travel costs.

Funds were managed through OpenCollective (opencollective.org), enabling transparent transactions for conference expenses. Publicly available records covered material costs, break refreshments, lunch catering, and the plenary dinner. Travel grants for plenaries and students were prioritised, with eligibility criteria set (valid student status and conference presentation) to support those lacking institutional funding, especially pre-PhD students.

### Website and Discord

Our website (issabc.org) was the central hub for disseminating relevant information before, during and after the conference, together with dedicated Discord channels (discord.com). Through these, we also achieved various other functions:

(1) **Marketing:** Before the conference, we used it to advertise the event, showcasing the location, speakers, and sponsors.
(2) **Centralised information:** The Attendee Hub (available only to registered attendees) contained schedules, the program, FAQs, and advice on the venue and the city.
(3) **Conference recordings:** The Attendee Hub also provided access to the Zoom links and talk recordings after the conference.
(4) **Science communication:** A blog facilitated science communication, allowing attendees to write blog posts to share their passion for the subject.

We suggest a structured website and active Discord promotion during registration to increase the visibility of the conference and the engagement of participants.

### Sustainability

International **c**onferences wield a notable environmental impact, consuming resources and generating waste. The third ISSABC prioritised sustainability for several reasons:

(1) **Environmental impact:** Sustainable practices, like reducing paper usage, adopting eco-friendly packaging, and minimising materials, cut the carbon footprint and waste.
(2) **Climate change mitigation:** Conference travel, especially international, leads to a substantial carbon footprint (Spinellis and Louridas, 2013). Strategies like offering virtual attendance options and choosing locations with good public transport mitigate travel-related emissions.
(3) **Economic efficiency:** Lowering energy and water consumption, and opting for sustainable catering can reduce costs. Avoiding unnecessary expenses, like conference gift bags, ensures financial viability and directing funds to more effective uses, such as student grants.
(4) **Ethical and social responsibility:** Embracing sustainability sends a message of caring for the planet. Avoiding unnecessary waste and choosing sustainable options demonstrate a commitment to ethical and social responsibility.
(5) **Innovation and inspiration:** Sustainable conferences showcase innovative solutions in eco-friendly technologies and practices, inspiring attendees to implement similar strategies in their organisations.
(6) **Long-term viability:** As environmental concerns grow, conferences not embracing sustainability may face declining participation. Promoting sustainable practices ensures long-term viability, considering worldwide travel disruptions like the COVID-19 pandemic.
(7) **Networking and collaboration:** Sustainable conferences encourage collaboration through initiatives like carpooling or ridesharing, fostering networking opportunities that extend beyond the conference itself.

In conclusion, encouraging sustainability in conferences is essential for minimising environmental impact, aligning with the ethical values of participants, reducing costs, and ensuring the long-term viability and success of these events. It not only benefits the environment but also enhances the overall conference experience and its positive impact on society. As such, some of the measures taken include:

(1) Hybrid participation option.
  - Increased attendance and reduced travel needs.
  - Provided online networking platform.
(2) No single-use plastics.
  - Provided reusable terracotta cups for the whole event. They also served as souvenirs for the participants, as they were traditional Mexican mugs, hand-made and purchased locally.
  - Catering service used no single-use plastic.
  - Name tag covers were requested back, to be reused.
(3) Encourage sustainable travel options, particularly for the grantees.
(4) Vegan and locally sourced catering options.
(5) Location with easy access to public transport.
(6) No printed programmes for the participants (online programmes were accessible by QR codes instead).

## Conference

### Conference format

The third ISSABC at UNAM spanned 3 days, emphasising accessibility via public transport. Presentation options included standard talks (10 min), flash talks (3 min), or posters. Five post-doctoral plenary speakers aimed for a balance between experience and relatability.

Presentations, in-person or virtual, allowed pre-recorded talks to account for unstable connections or time zone issues, with the choice of English or Spanish. Live-streamed and recorded talks on Zoom accommodated time zone differences. Discord channels facilitated discussions for each session, plenary, and poster.

Digital posters on the conference website and Discord allowed for online questions. A 360-degree camera provided an in-person-like view for virtual participants. Three workshops and a career development roundtable were broadcast to virtual attendees. The event concluded with the awarding of five prizes for the best talk, best poster, and best flash talk, along with three honorary mentions. This not only contributed to the sense of community but also boosted participants’ confidence in the contributions to the future of the field (for details on winners, see Supplementary Materials).

The round-table discussion, guided by organisers, covered mental health, discrimination, and career development. Postdocs, including plenary speakers, addressed audience questions. The event's significance lies in bridging the gap between senior researchers and students, emphasising discussions across various career stages and experiences. Such discussions at ISSABC contribute to a supportive ECR community, fostering resource-sharing beyond sessions. For instance, addressing post-doc challenges led to shared post-doc lists, exemplifying the valuable collaborative nature of these panel discussions.

### Attendance

**Fig. 2.**
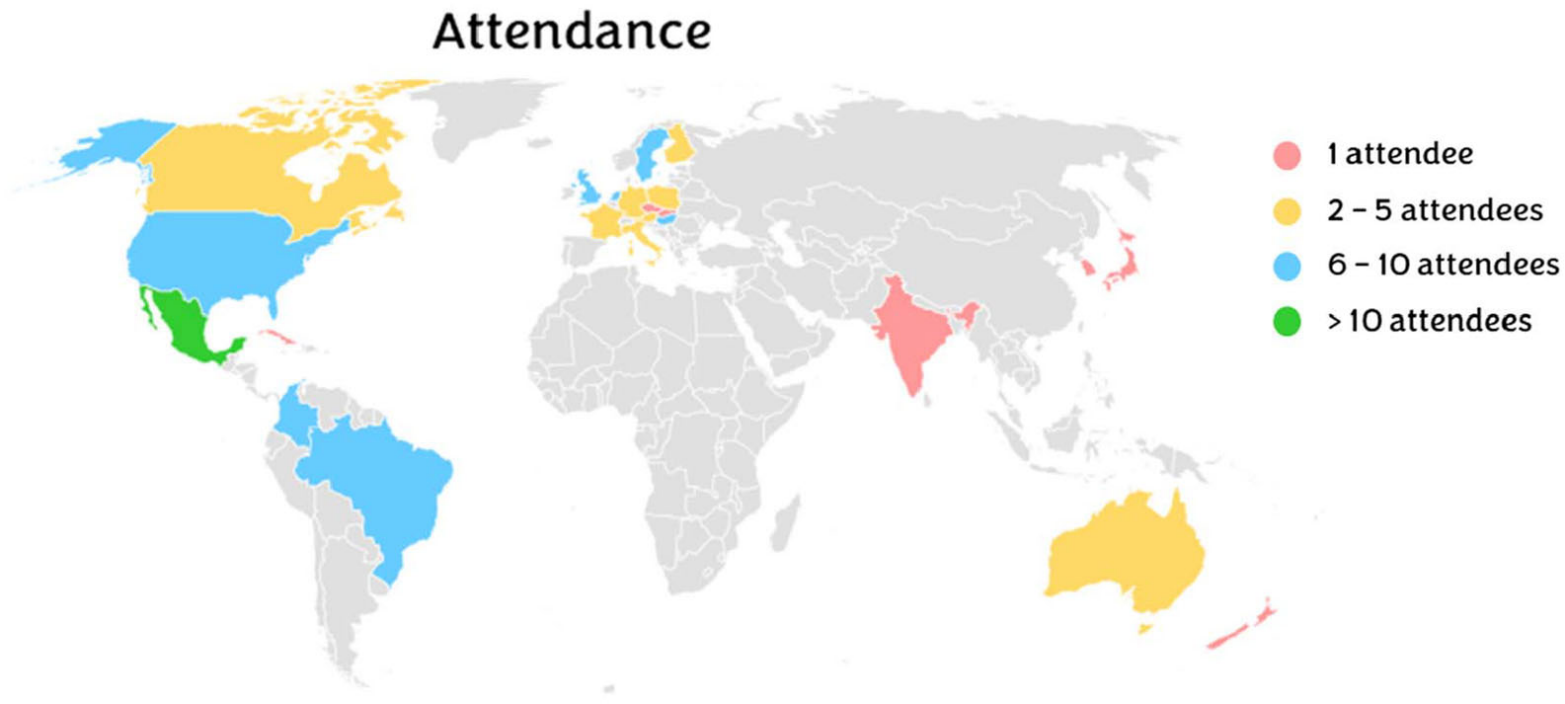
**Geographical origin of the home institution of the participants.** The red colour reflects the countries where there was only one attendee; yellow reflects when there were two to five attendees; blue when there were six to 10 attendees; and green when there were more than 10 attendees at the conference from that country.

The event was attended by 101 participants in person and 79 virtually: representing a total of 24 countries (Fig. 2). Most of the attendees were students (81%), and more than 62% of participants identified as female, followed by male (31%), non-binary (3%) and non-disclosed (4%). The dual-language nature of the event was beneficial for the participants, as 53% of participants only spoke one of the two languages (only Spanish: 16%, only English: 37%, both: 47%).

Several students showed interest in attending the event but were unable to do so mainly due to the cost of travel. Moreover, some of the ISSABC grantees were forced to reject their grants due to administrative visa issues. We would like to highlight the need to approach participants who require visas as early as possible and provide them with the necessary certificates. Unfortunately, even with a timely approach, some bureaucratic hurdles remain insurmountable.

In total, we had 69 standard talks (45 in person, 24 online), 12 flash talks (five in person, seven online) and 29 posters (18 in person, 11 online), in addition to the five in-person plenary talks.

### Emerging field

During the third ISSABC, the incorporation of animal welfare into animal behaviour research emerged as a focal point of interest among ECR participants. Numerous talks delved into this theme, and the workshop dedicated to the topic reached full capacity.

### Testimonials

“*I wanted to express my astonishment at your exceptional work during the recent conference. The experience was truly incredible and immensely valuable for my personal and professional growth*”- MSc student from Colombia.

“*First, I would like to thank you for organising the conference! Also, thank you for making it a hybrid event, which allowed participation for those who were unable to afford travel expenses*” - PhD student from Hungary.

## Recommendations

- **Local representation** greatly facilitated initial conference planning with a committee member studying in Mexico. This established a robust communication foundation with the host university, crucial for overcoming language barriers and establishing trust. However, when the representative withdrew for personal reasons, communication challenges ensued.
- **Contingency plan.** For the current conference, a virtual option served as a contingency plan for potential sponsorship shortages or unforeseen issues. This allowed remote participation and presentation, ensuring flexibility in the face of challenges.
- **Plenary speakers you can rely on.** A rigorous interview process for plenary speakers, particularly lesser-known postdoctoral researchers, was crucial for the third ISSABC. Pre-selecting candidates based on personal connections and nominations, we sought diverse career paths and study systems. Establishing good relationships with speakers was also vital for handling last-minute changes or session chairing in smaller meetings.
- **Good marketing strategy.** An early and impactful marketing campaign with a professional logo, event name, and an appealing website is paramount. Utilising social media, in-person conferences, and established scientific societies is crucial, tailoring efforts to the target community's preferred channels (e.g. Facebook and Instagram for Mexican and Latin American students).

## Future steps

Events such as the one described can build credibility and impact for the presenters, attendees and organisers even when the main event has come to an end. This can be achieved by ensuring continuity and credibility over the subsequent editions of the conference. We emphasise the importance of clear communication between the organisers of past and future conferences, not only on their successes but also on the mistakes that have been made. Especially when preparing student conferences, where most organisers are by nature inexperienced, such information transmission can be highly beneficial. One of the main objectives of the event for the current organisers was to offer a sense of community to the participants. To provide this after the event, the ISSABC aims to offer continuous opportunities to students to present and share their work and connect with other researchers in the field. Through future editions of the conference, we aim to inspire the next generation of animal behaviour researchers to view conferences as barrier-free opportunities to share knowledge and their passion for science.

## Supplementary Material

10.1242/biolopen.060290_sup1 Supplementary information
