# Supplementary material for: Beyond barriers, towards diversity: how hybrid student conferences can drive accessibility
Source: Biol Open. 2024 Jan 30;13(2):bio060290. doi: 10.1242/bio.060290 (PMC10855212; doi:10.1242/bio.060290)
Supplement: Supplementary information [file biolopen-13-060290-s1.pdf]

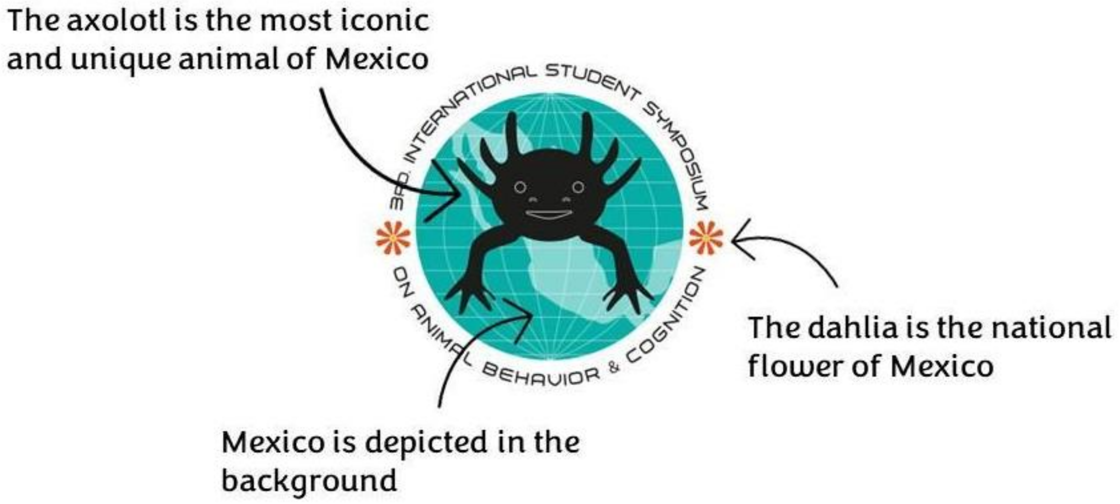

Fig. S1. Logo designed by collaborators at UNAM for the 3rd International Student Symposium on Animal Behaviour and Cognition.

Winners of posters and talks

Best posters

- Spanish: Cassandra Rubio, " La casa adecuada: Preferencia de refugio del bagre de canal", UNAM
- English: Sierra R. Lissick, " Reaching new heights: Suspended foraging boxes may benefit giraffes in zoological facilities", University of Alaska Southeast

Best flash talk:

- Kata Vékony, " The ball is mine! Validation of the dog rank questionnaire! Eotvos Lorand University, Hungary

Best talk:

- Spanish: Selene Asiul Barba Bedolla, "Cuidado parental de *Ara militaris*", Universidad Michoacana de San Nicolás de Hidalgo
- English: Rachel Layton, "Behavioural-based pig management and training in infectious disease research", CSIRO Australian Center for Disease Preparedness/ University of Melbourne

Runner-ups:

- Spanish: Maribel Rojas Montoya, "Diestro o Izquierdo: ¿Será que el microhábitat, el tamaño corporal y tipo de locomoción están relacionados con el nivel de lateralidad de las extremidades anteriores de los anuros?", Universidad del Quindío
- English: Stephanie Ruck "Response of the Western Fence Lizard (*Sceloporus occidentalis*) to Post-Fire Disturbances", California State University, San Bernardino
- English: Océane Liehrmann, "Measuring reindeer emotional changes using infrared thermal imaging", University of Turku

Website

There were a few main functions the website had:

1. Marketing: Before the conference, the website functioned as the primary advertisement for the event, showcasing the location, plenary speakers' announcements and sponsors who had signed up so far.
2. Centralised Info: The website allowed us to collate all the relevant information for the attendees in one place. Our Attendee Hub (Fig. 3) contained schedule timings, an updated programme, an FAQ and a forum. It also provided a place for useful secondary information, such as things to do, safety guidance and a platform to host our photography competition. This reduced workload by mitigating recurring questions.
3. Linking: We used the website to link to our various other platforms, for example, our Discord group and social media pages, as well as the Zoom links to the live talks for the online attendees to access.
4. Conference Recordings: After the conference, the Attendee Hub remained an ideal location to provide access to the talk recordings so attendees could rewatch or catch talks they had missed.
5. Point of Contact: Finally, the website had a built-in contact form which made getting in contact with our committee easy for anyone who had any questions.
6. Science Communication: The blog area allowed us to create a place for science communication and engagement, as we could invite attendees to write articles about their research or topics of interest to be read by the wider community.

Alongside our website, we used Discord - a community-based messaging app similar to Slack, as a moment-to-moment digital companion. The primary uses we found for Discord were:

1. Instant Messaging: The quick-messaging format that Discord takes enabled us to make announcements and update schedule timings on the go, allowing us to pass information on to attendees live. It also allowed us to quickly answer questions from attendees who may not be able to locate us in-person or who are online.
2. Networking: The app also provided a safe environment for attendees to network and discuss talks, with each talk session having a separate channel attendees could access.
3. Posters: Each poster presentation had a dedicated channel, and we could provide an online link to the posters and a space for presenters to answer questions from attendees who were not in the same location (i.e. in-person or online) as them.Our website was well used by visitors from North America, Central and Western Europe, and Australia (Fig 4). To a lesser extent, it was also accessed in some parts of Asia and South America, however, there were very few pageviews from Africa, Southeast Asia, Eastern Europe or the Middle East. It should be noted that the data in Fig 4 is only representative of the most active 8 countries each month. When we look at the Attendee Hub in particular, we see a circa 6 times increase in usage activity during the conference month, at nearly 2000 pageviews, which drops off in the following months. Our Discord server was only accessed by approximately one-quarter of the conference attendees (n=30, excluding organisers), of which half (n=16) actively used it. Of these attendees, most use was made during the poster presentations, with the majority being online attendees.

Biology Open • Supplementary information

Table S1. Comparison of different methods of audience engagement

Platform	Website	Discord	Slack	Social Media
Access	Can create public and private areas of the website to control who sees what. Centralised and easy to access.	Must be a part of the community/channel to access any information. Less common in scientific communities.	Must be a part of the community/channel to access any information. More common in scientific and professional communities.	Usually requires an account to access, but accessible by anyone, not just community members.
Efficiency	Slower at information transfer	Instant-messaging	Instant-messaging	Quick info transfer and easily shareable, so effective at expanding audiences, but may take time to create the media
Longevity	Information remains and is prioritised however you want	Messages remain and may get difficult to find in long conversation chains	Messages disappear after a set period. Can be extended with a subscription	Information remains but may get lost among other posts, accounts and platform algorithms
Flexibility	Very flexible functionality, but requires technical knowledge	Can be used for messenger, FAQ/forum, and voice call hosting. Also, video hosting, but requires paid tier for a larger audience	Primarily instant messaging	Dependent on platform
Awareness	Requires email to effectively notify of updates	Instant mobile notifications	Instant mobile notifications	Instant mobile notifications

In Table 1 we have summarised the key benefits and drawbacks associated with different methods of attendee engagement. While the website serves as a good, centralised hub, with flexible functionality, it is a slower method of information dissemination. Discord, for instance, excels in providing immediate responses and notifications better than traditional website or

Biology Open • Supplementary information

email contact. It also proves superior in hosting forum-style discussions, rendering the website's forum somewhat redundant after Discord's introduction. Discord's long-term message record, offered for free, is another advantage over platforms such as Slack. While it boasts video hosting capabilities that could potentially replace Zoom, it lacks native recording functionality and may require paid boosting of channels for larger audiences. However, the low adoption rate of Discord among attendees is worth noting. This is possibly due to the relative unfamiliarity compared to business-orientated apps such as Slack. In addition, there is the requirement of downloading an application and creating an account which was not necessary for the website (where accounts were pre-made for all attendees). In hindsight, we should have actively promoted Discord's uptake during the registration process and earlier on in the development of the conference. We propose a mix of a structured website that offers customizability and a dedicated platform to host more interactive discussions, aiding the interaction of online and in-person participants.

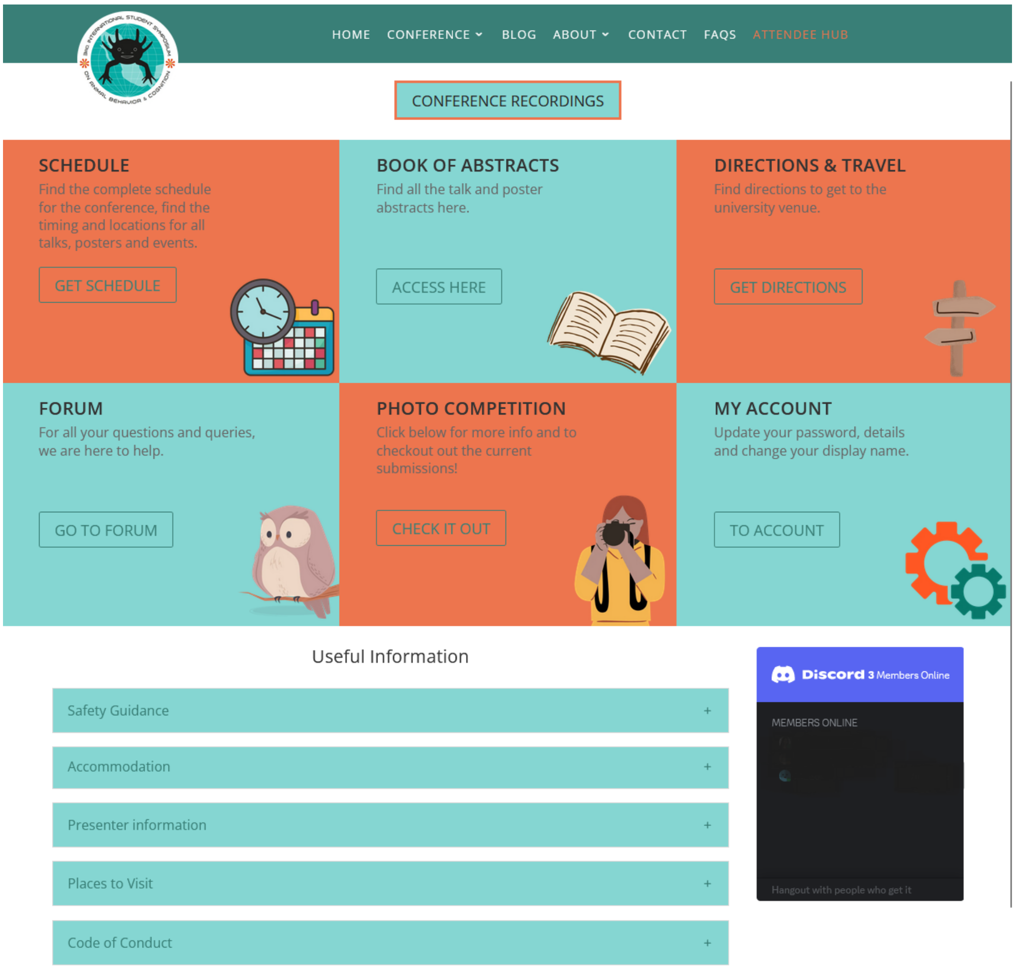

Fig. S2. A detailed view of the issabc.org website attendee hub.

Biology Open • Supplementary information
